# A Mid-Term Result of the Treatment of Intra-Articular Calcaneal Fractures with the Use of Intramedullary Nailing

**DOI:** 10.3390/jcm14041369

**Published:** 2025-02-19

**Authors:** Piotr Sypien, Dariusz Grzelecki

**Affiliations:** 1 Department of Trauma and Orthopedic Surgery, Sebastian Petrycy Health Care Facility, Szpitalna 1, 33-200 Dabrowa Tarnowska, Poland; 2 Department of Orthopedics and Rheumoorthopedics, Centre of Postgraduate Medical Education, Professor Adam Gruca Orthopedic and Trauma Teaching Hospital, Konarskiego 13, 05-400 Otwock, Poland

**Keywords:** calcaneal fracture, close reduction internal fixation, CALCAnail, postoperative complications

## Abstract

**Background:** Intra-articular calcaneal fracture (CF) treatment is associated with a high risk of complications, but closed reduction and internal fixation (CRIF) is a minimally invasive alternative for treatment. **Methods**: Forty-eight patients treated with CRIF and CALCAnail^®^ due to intra-articular CF between 2016 and 2021 were analyzed to check union time, complication rate, and functionality after the intervention. Functional and pain outcomes were assessed, including the Maryland Foot Score (MFS), American Orthopedic Foot & Ankle Society (AOFAS) scale questionnaires, and the numerical pain scale (NRS) at mid-term follow-ups 2–5 years after the intervention. **Results:** Intervention increased median Böhler’s angle from 21.5° to 32° (*p* < 0.01). The median bone union time was 12 weeks. The risk of malunion was higher in patients with Sanders type 4 (RR = 2.28; 95% CI 1.11–4.72) and those operated on later than the 2nd day after injury (RR = 2.1; 95% CI 1.08–4.09). Patients with at least one of the comorbidities (nicotinism, diabetes, obesity) had a higher risk of intensive pain (NRS > 3) 2–5 years after surgery (RR = 1.69; 95% CI 1.06–2.68), and 84% were satisfied with their treatment. Other complications included complex regional pain syndrome in two patients (4%), malunion in three (6%), and surgical site infection in two (4%). The MFS had a median score of 85 points, while that of the AOFAS was 82 points. **Conclusions**: CRIF, with the use of the CALCAnail^®^ implant, allows doctors to restore anatomical relationships around the subtalar joint, resulting in good clinical and functional results.

## 1. Introduction

Calcaneus fractures (CFs) are the most common tarsal injuries and account for about 2% of all fractures [1]. Most of them are the result of high-energy trauma, such as a fall from a height [2]. The energy released during the injury causes destruction and displacement of the entire bone and its articular surfaces, particularly damaging the subtalar joint. Therefore, up to 70% of CFs are multifragmented and intra-articular [2].

Imaging studies are the cornerstone of the diagnosis of CF. On the X-ray of the foot in lateral projection, an angle between the line joining the highest point of the anterior process of the calcaneus and the highest point of the posterior articular facet with the highest point of calcaneal tuberosity is known as the Böhler angle (BA). Under normal anatomical conditions, the BA is about 25–40 degrees. Reduction of the BA is an indicator of CF severity, while restoration of the BA during surgery is a correlated functional outcome [3].

The use of computed tomography (CT) allows us to understand the accurate fracture morphology and assess severity of the damage of the articular surface of the subtalar joint. Currently, the most widely used CT classification for CF is Sanders classification, which is based on the number of intra-articular fracture lines and their location [4]. This ranges from type I with nondisplaced CF, followed by types II and III with different combinations regarding the number of significant fracture lines, to type IV, in which there are combined fractures with displacement of the articular surface of the subtalar joint by more than 2 mm. Sanders’s classification is beneficial not only for understanding typical CF patterns but also for predicting outcomes. Particularly prone to treatment failure is type IV, according to Sanders, which, due to significant displacement, affects the development of posttraumatic arthrosis [4].

Due to the complexity of CF, it is still difficult to establish proper management of this type of injury, and its treatment poses serious clinical challenges for orthopedic surgeons. Conservative treatment is burdened with a high risk of malunion caused by complex anatomy, complicated articulations, insufficient soft tissue coverage, and unreconstructed anatomic relations, which can permanently induce changes in the biomechanics of the foot, favoring prolonged pain syndromes and the development of degenerative changes [5,6].

Several surgical treatment methods have been introduced so far to reduce and fixate CF properly. Recently, open reduction and internal fixation (ORIF) with the use of low-profile anatomic locking plates has become the preferred method of intervention for CF, but it requires an extended skin incision, which is associated with a long wait for appropriate surgery and a high risk of complications from adjacent soft tissues, such as surgical site infection (SSI) or necrosis of the wound edges, whose percentage can be as high as 30% [5,7]. The use of minimally invasive procedures is an alternative and a promising treatment option that may reduce postoperative complications. Among the methods used are interventions with indirect fracture reduction and fixation with a minimal skin incision, such as cannulated screws or K-wires [2,5,8]. In addition, minimal skin incisions are made using access reduction and plating from the sinus tarsi, which significantly reduces soft tissue traumatization [9].

One of the minimally invasive surgical methods used to treat CF is closed reduction and internal fixation (CRIF) with the use of intramedullary nailing (IM). With this method, it is possible to limit the degree of extensive surgical interference as in ORIF. In turn, it has the advantage of providing stability to the fracture, greater than other minimally invasive treatments such as K-wires, and avoiding prolonged immobilization in a plaster cast [5,6,7]. However, there is still insufficient data assessing CRIF with the use of IM, describing the results, complications, and benefits for the patients. Few studies show satisfactory long-term results of CRIF in CF [10,11]. Their functional scores average more than 80 on the AOFAS scale, which has been validated in the literature as a good result [12]. However, these results are limited and require further analysis. This retrospective study aims to evaluate mid-term radiological, clinical, and functional outcomes of the treatment of intra-articular CF with IM.

## 2. Materials and Methods

### 2.1. Study Design

**Figure 1:**
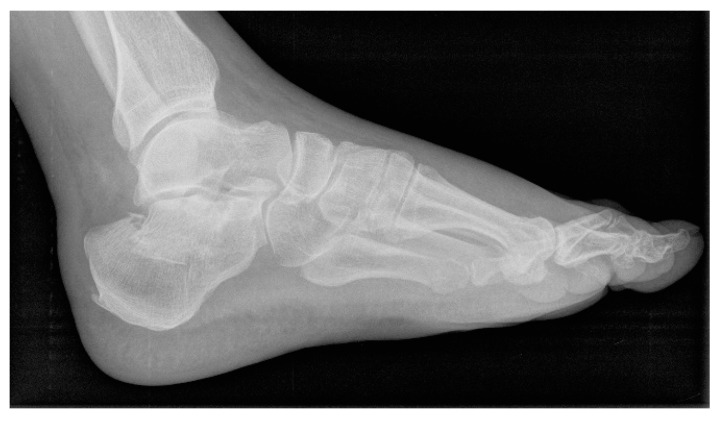
Lateral view radiograph of a calcaneal fracture.

**Figure 2:**
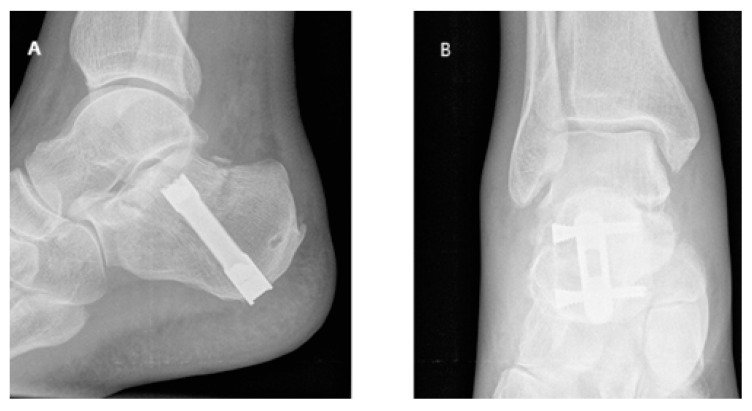
CRIF with CALCAnail^®^: (**A**) lateral and (**B**) anterior–posterior (AP) radiographs.

This research was carried out in our hospital to evaluate the mid-term outcomes of CRIF with IM (CALCAnail^®^, FH Ortho, Chicago, IL, USA) in treating intra-articular CF (Figure 1 and Figure 2). This retrospective observational study included forty-eight patients (36 men and 12 women) treated between 2016 and 2021. The median age was 57 (interquartile range, IQR = 48.75–64), and the median body mass index (BMI) was 26.35 kg/m^2^ (IQR = 23.97–28.15). The study included hospital stays with surgery and follow-ups 2–5 years after intervention.

### 2.2. Data Collection

Data were collected from medical records, radiological examinations, and during outpatient visits. During the study, 67 patients were treated in the department for CF. The main inclusion in the study criterion was intra-articular CF. Additional inclusion criteria were available: preoperative and postoperative imaging data and complete follow-up data, which 48 patients finally met. The exclusion criteria were extra-articular CF or nondisplaced intra-articular CF (4 patients), disqualification due to general contraindications to surgical treatment (2 patients), another method of surgical treatment of CF (6 patients), C-arm breakdown in the operating room (1 patient), duo-lateral CF (1 patient), refusal of surgical treatment (2 patients). To avoid selection bias, CALCAnail^®^ was the surgical technique of choice used in our department at the time of the research.

The demographical and clinical parameters analyzed in this study were gender, age, time and circumstances of injury, length of surgery and day of intervention after injury, comorbidities, functionality after treatment, and overall satisfaction with treatment. Obesity was recognized when the BMI was higher than 30 kg/m^2^. Each patient received whole diagnostic imaging. Pre- and postoperative X-rays of the foot were performed, and we measured the BA twice for each patient. A value of 25–40° was assumed as normal BA. The position of the longitudinal axis of the implant relative to the line joining the highest point of the anterior process of the calcaneus and the highest point of the posterior articular facet was evaluated in post-operative X-rays, and the value of 90 ± 5 degrees was assumed to be correct. Every patient also underwent a CT before surgery, and the Sanders classification was used to determine the severity of the injury [4]. The Maryland Foot Score (MFS) and the American Orthopedic Foot & Ankle Society (AOFAS) scale were used to measure functional outcomes after the treatment [5,13]. A score of more than 80 points was considered a good result [14]. In addition, each patient was asked to give a subjective assessment of satisfaction with the treatment provided. A numerical rating scale (NRS) was used to measure pain.

### 2.3. Treatment Method

Initial treatment for all participants was bed rest, analgesia, elevation, and nonbearing of the foot.

CALCAnail^®^ was used in the definitive treatment for each patient. The procedure was performed under spinal anesthesia. Each patient received 2 g of cefazoline in one dose (3 g > 120 kg body weight) intravenously for preoperative antibiotic prophylaxis administered 30 min before the intervention.

**Figure 3:**
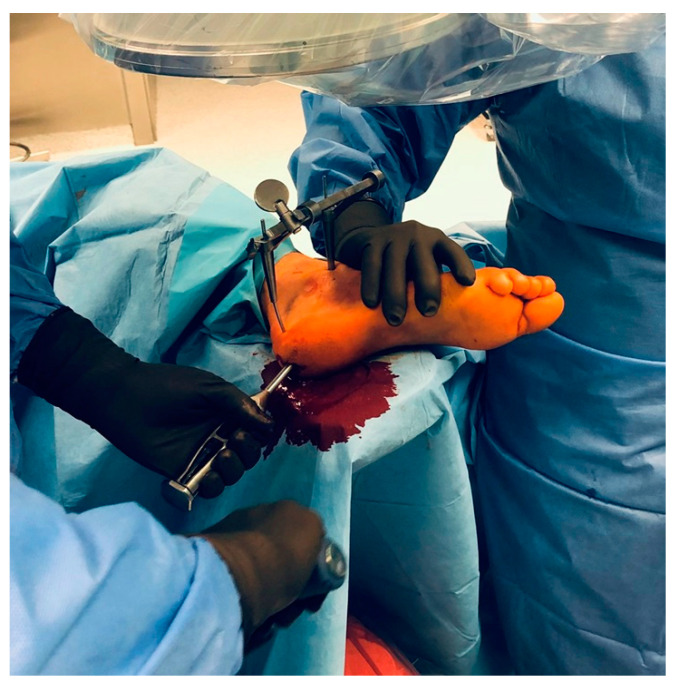
Restoration of the articular surface of the subtalar joint under fluoroscopy using a pusher through the working channel in the calcaneal tuberosity.

The patient was positioned in the lateral decubitus position during the surgery. Two Steinmann pins were inserted under fluoroscopy into the calcaneal tuberosity and talar bone. The fracture was reduced by a distractor implanted into the Steinmann pins, and the BA was restored. A small, 2 cm longitudinal incision was made distally into the Achilles tendon, and a working channel in the calcaneal tuberosity was created. A pusher through the working channel was then used to restore the articular surface under fluoroscopy of the articular surface of the subtalar joint. Also, through the working channel, the CALCAnail^®^ was inserted to fix the CF and lock it with 2 screws percutaneously (Figure 3).

Each patient was immobilized after surgery in a plaster splint for 2 weeks. Patients received standard anticoagulant prophylaxis with enoxaparin 40 mg/0.4 mL subcutaneously unless a change was necessary due to other indications. A postoperative radiograph was repeated the day after surgery and during follow-ups. The surgical wound was cleaned every 3 or 4 days until the sutures were removed, approximately 2 weeks after intervention. After immobilization, the physiotherapist encouraged patients to exercise their toes and ankle joints. Crutch-assisted walking was allowed two or three days postoperatively. Full weight bearing was initiated 3 months after surgery.

### 2.4. Statistical Analysis

Statistical analyses were conducted using Microsoft Excel 16^®^ (Microsoft Corporation, Redmond, WA, USA) and PQStat 1.8.6^®^ (PQStat, Poznan, Poland). Categorical data were summarized using frequency (n), percentages (%), mean, standard deviation (SD), median, and IQR. The Shapiro–Wilk test was used to check the normality of the distribution. The Student’s *t*-test was used for parametric variables, while the Mann–Whitney U test was used for nonparametric variables. The Fisher test and risk ratio (RR) were used to check differences for dichotomous variables. *p* < 0.05 was assumed to be statistically significant, and RR was estimated with a 95% confidence interval (CI).

## 3. Results

Isolated fractures dominated, although 10 patients (21%) had additional injuries such as ankle fractures (3), spine fractures (4), forearm fractures (1), knee sprains (1), or open head wounds (1). Almost all injuries were high-energy, and a fall from a ladder was the most frequent (Table 1). The median BA at admission was 21.5 degrees (IQR = 17–27).

**Figure 4:**
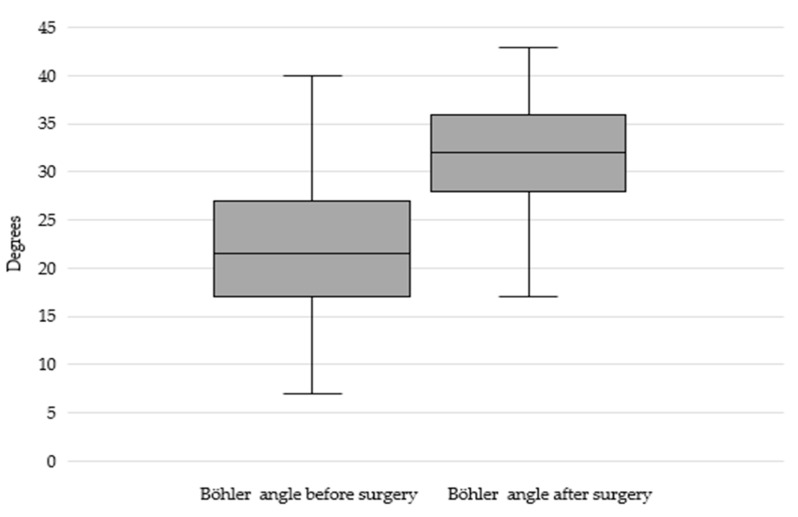
Change in BA before and after intervention (in degrees); *p* < 0.01.

The average hospital stay was 4.6 days (SD = 2.17). Surgery was mainly performed on the second day after the injury. The median operation time was 40 min (IQR = 30–46). The surgical intervention improved the median value of BA correction to 32° (IQR = 28–36; *p* = 0.02, Figure 4). It resulted in 45 patients (94%) achieving a correct calcaneal angle range and adequate fracture reduction.

**Figure 5:**
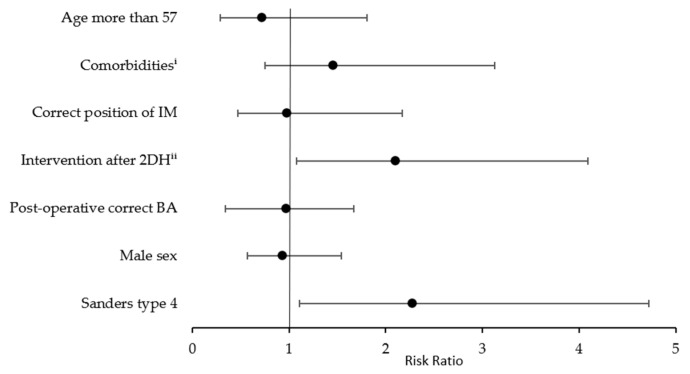
Risks of prolonged bone union time (RR with CI). ^i^ Comorbidities: obesity, nicotinism, or diabetes. ^ii^ Intervention performed later than on the 2nd day after injury.

Follow-up continued for approximately 7 months, although in nine patients (17%), it continued for more than 35 months. The time of bone union varied with a median of 12 weeks (IQR = 9–16.25). In nine patients (18%), the union was observed more than 20 weeks after intervention (Table 2). Patients with Sanders type 4 fracture and with intervention performed later than on the second day after the trauma presented a higher risk of prolonged union time (Figure 5). In addition, statistically significantly longer union time was seen among patients older than 57 (24.00% vs. 48.15%; *p* = 0.04) and nearly statistically significantly more frequently among women (57.14% vs. 29.73%; *p* = 0.07). There was no effect on healing bone time by the presence of at least one of the comorbidities (nicotinism, diabetes, obesity; *p* = 0.43).

**Figure 6:**
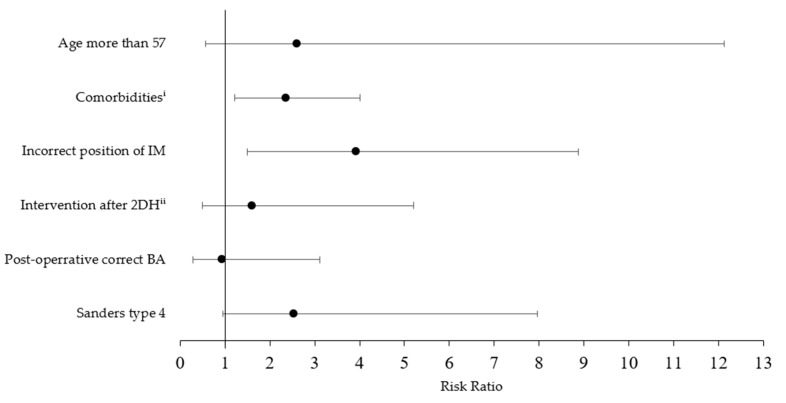
Risks of complications for CF after intervention (RR with CI). ^i^ Comorbidities: obesity, nicotinism, or diabetes. ^ii^ Intervention was performed later than on the 2nd day after the injury.

Complications observed during the next ambulatory treatment included complex regional pain syndrome (CRPS) in two patients (4%), malunion in three (6%), and surgical site infection (SSI) in two (4%, Table 3). Patients with misaligned fixation had a higher risk of complication (Figure 6). In malunion, two patients (66%) required corrective osteotomies. After a second intervention, one of the patients achieved relief from her complaints, but this was associated with an extended union time of up to 37 weeks. The second one is still experiencing significant restriction of foot mobility. This patient had a baseline BA of 7 degrees and type VI according to Sanders’s classification. He received further treatment—a corrective osteotomy of the calcaneus and then triple arthrodesis (hindfoot fusion). All patients with CRPS received intensive rehabilitation, including cryotherapy, laser therapy, cooling baths, TENS transcutaneous nerve stimulation, and manual therapy. In the end, two of them required steroid injections. Complaints were noticeably decreased after interventions and were clinically irrelevant or acceptable to the patients. Removal of the fixator and intravenous antibiotic therapy based on microbiological testing healed inflammation in SSI. In the two cases, the union was observed after 8 and 10 months.

At follow-ups between 2 and 5 years after the intervention, up to 37 (77%) patients complained about pain in the operated foot, although 32 (67%) only occasionally. Postoperative pain was higher among women (53.33% vs. 21.62%, *p* = 0.02), patients operated on later than the 2nd day of hospitalization (29.28% vs. 20.83%, *p* = 0.03), and those who have at least one comorbidity (nicotinism, diabetes, or obesity; 39.29% vs. 20.83%, *p* = 0.04). Sixteen (33%) described the intensity of pain as NRS > 3, and fourteen (29%) used at least occasional pain medication. Some functional limitations, such as limping or reduced range of motion, were observed in 25 cases (48%).

The MFS median score was 85 points (IQR = 77.5–95) and was independent of gender (71.43% female vs. 67.57% male, *p* = 0.79), age (over 57, 70.83% vs. 66.67% under 57, *p* = 0.49), and comorbidities (obesity, nicotinism, or diabetes; 70.37% vs. 66.67%, *p* = 0.77). On the other hand, it was significantly worse in the group with Sanders type 4 fracture (50% vs. 75.68%, *p* = 0.06) and significantly more frequent in those who received surgery on the second day of hospitalization (78.79% vs. 50.00%, *p* = 0.04). The AOFAS resulted in a median score of 82 points (IQR = 73–91.25). Overall, 40 (84%) patients are satisfied with treatment.

## 4. Discussion

The calcaneus is the primary weight-bearing bone in the foot, which makes it vulnerable to injuries. The primary cause of CF is high-energy trauma, such as a fall from a height [1,9,14]. This destroys the articular surfaces of the subtalar joint and results in a high risk of developing posttraumatic arthrosis. Conservative treatment perpetuates bone deformities and worsening functional outcomes [5]. In addition to fixation, surgical treatment aims to restore anatomical relationships of the subtalar joint. A substitute for the amount of energy absorbed by the foot is BA [2]. It is also considered a reliable tool for assessing the severity of injury and is widely used in analyzing the outcome of surgical treatment [2,3]. Importantly, proper restoration of the articular surfaces affects the functionality of the foot at long-term follow-ups [3,5]. Surgical treatment, moreover, is better than conservative in functional results, increasing the range of motion of the operated foot [5]. Open and closed treatment techniques allow for comparable restoration of the BA [8,9,15]. Using a distractor and a pusher through the calcaneus’s working canal allows the shaft’s elevation and restores the articular surface during CRIF [10,16]. The use of bone grafts does not significantly affect treatment outcomes [17].

The appropriate timing of surgery for the fracture is still a matter of debate, as soft tissue swelling coexisting with injury can significantly affect the time and quality of intervention. Zhang et al. indicated no beneficial effect at the ORIF of the day of intervention on the amount of blood loss and postoperative drainage [18]. Similarly, Kim et al. showed no differences in relation to clinical results between early intervention and staged treatment with a transient external stabilizer [19]. On the other hand, ORIF using a plate from the typical lateral extensive approach involves performing the intervention most often 1–3 weeks after the injury [6]. However, clinical data on the timing of the intervention and its impact on healing are limited. This seems to be less important when using minimally invasive approaches. A previous study on CALCAnail^®^ pointed to good clinical results with intervention carried out on an average of the 6th day after injury [16]. In our research, treatment was performed earlier, and comparable functional results were achieved in mid-term follow-up. In addition, we have noted the benefits of rapid reduction of the fragments and stabilization before swelling builds up, as this reduces the risk of complications. Early minimally invasive intervention reduces soft tissue traumatization and is acceptable immediately [20]. Additionally, the day of the surgery after an injury did not affect the length of the intervention. However, the risk of developing transient pain syndrome after an earlier intervention is noteworthy, which still requires thorough studies.

Soft tissue complication rates of up to 30% are a significant complication in the treatment of CF [15]. Additional traumatization is associated with open reduction regardless of the surgical approach [21]. Minimally invasive methods reduce the risk of SSI. The rate of SSI in our study was similar to that of another paper evaluating the CRIF of CF [11]. Low soft tissue traumatization in minimally invasive treatment decreases the occurrence of adverse events and reduces hospitalization time [22]. The possible incomplete positioning of the set-offs in CRIF might cause malunion and remains controversial. In our study, a malunion was recognized in 6% of patients, which is comparable to other studies [23,24]. This complication is a debilitating condition due to severe hindfoot deformity with painful arthritis of the popliteal joint, excessive overloading of adjacent joints, soft tissue imbalance, and compression [25]. Surgical treatment can include corrective osteotomy with partial removal of the malformed lateral wall of the calcaneus from a lateral approach, which can increase the range of motion and significantly reduce pain [24,26].

In long-term follow-up, patients achieved acceptable results, with the average union time being slightly longer in comparison to other minimally invasive methods [9]. For some participants, long-term implications of the injury included limited mobility, although the vast majority only slightly, and it does not significantly affect functionality. Fascione et al., in their clinical evaluation of the results of CALCAnail^®^ treatment in CF, obtained a mean AOFAS score of 85 points, which is comparable to our study and satisfactory [27]. Also, Kumar et al. show excellent functionality after minimally invasive intervention in CF, which is significantly better than after ORIF [15]. In addition, the use of ORIF with a plate is often associated with the need to remove hardware due to persistent postoperative pain, which is indicated as the most common indication for reoperation after CF [3,4,5]. On the other hand, the use of IM in CF provides BA restoration and functional results comparable to other minimally invasive methods [8,21].

Functional problems are particularly concerning for patients with a type IV fracture, according to Sanders’s classification, due to the complexity of the clinical presentation (multiple bone fragments and more than 2 mm of articular displacement). This leads to poorer outcomes and an increased risk of developing osteoarthritis [5]. Operative treatment may not effectively restore the subtalar joint’s articular surfaces, especially when using indirect methods like CALCAnail^®^. These patients, in spite of treatment, have significantly worse functional outcomes after orthopedic treatment in comparison to other stages, according to Sanders’s classification, leading to osteoarthritis [3]. In order to reduce complications of this type, it is recommended that primary subtrochanteric arthrodesis be performed, as well as IM during CRIF [10]. Even if subtalar arthrodesis becomes necessary, patients will benefit from primary anatomical reconstruction of tarsal geometry, as in situ arthrodesis is associated with better outcomes than corrective arthrodesis of hindfoot deformities in malunion of CF [7,28]. A plate fixation gives a better chance of restoring the articular surface, but due to the complexity of the fracture, there are usually very poor local conditions for the operation, and the patient must wait to obtain a suitable soft tissue compromise [2,15].

The use of IM in CF provides good fracture stability and satisfactory biomechanical results [11]. For this reason, CRIF, in comparison to ORIF, is associated with a lower cost of treatment and a faster return to work [8,15]. Additionally, for the majority of patients, it creates optimal conditions for a return to sports [27].

Our study has several limitations that should be considered before analyzing results. First, this is a single-center study on a small group of patients. In addition, it is a retrospective observational study with no comparison to another method. However, there are still few studies in the available literature demonstrating the effectiveness of CF treatment using modern therapies or comparisons with other treatments, especially using IM. There is a need for reliable, multicenter studies accurately assessing the patients’ quality of life after the intervention and its comparisons to other surgical methods used. Mid-term follow-up shows that, after obtaining union, the most critical problems for patients are the presence of persistent pain and functional impairment. There is also a need for a long-term analysis of how this surgical method affects the frequency and rate of development of degenerative changes in the lower limb in the future.

## 5. Conclusions

The use of CRIF with IM allows the satisfactory recreation of anatomical relationships in the foot with minimal additional soft tissue traumatization, especially in type II and III fractures according to Sanders classification. Fixation with CALCAnail^®^ effectively improves the value of BA, and the minimally invasive approach reduces the risk of complications. Additionally, better outcomes are achieved among young patients and those operated on early. Due to its minimally invasive procedure, surgical treatment is possible in the early days after the fracture without a long wait for adequate soft tissue quality. Patients accept the intervention’s results, although it may be associated with prolonged bone union time and may be accompanied by the onset of chronic postoperative pain.

## Figures and Tables

**Table 1:** Characteristics of patients included in the research.

Parameter		Number	%
Circumstances of injury	Fall from a ladder	27	57.25
	Fall from stairs	10	18.25
	Fall from a bicycle	2	4.67
	Fall from a truck	1	2.08
	Jump into a pool	2	4.67
	Slip	6	13.08
Type of CF according to Sanders classification	II A	7	14.58
	II B	9	18.75
	II C	2	4.17
	III AB	10	20.83
	III AC	7	14.58
	III BC	5	10.42
	IV	8	16.67
Site of injury	Left foot	25	52.08
	Right foot	23	47.92

**Table 2:** Median union time of CF according to Sanders classification.

Type of CF According to Sanders Classification	II	III	IV
Median union time (weeks, IQR)	11 (10–15)	12 (9–16)	16 (12–19)

**Table 3:** Complications of CF according to Sanders classification.

Type of CF According to Sanders Classification	II	III	IV
Complex regional pain syndrome	0	1 (4.5%)	1 (12.5%)
Malunion	0	1 (4.5%)	2 (25%)
Surgical site infection	1 (5%)	0	1 (12.5%)
Total	1 (5%)	2 (9%)	4 (50%)

## Data Availability

For this study, we cannot publicly share individual data due to participant confidentiality. Qualifying researchers may apply for access to the minimal, anonymous data set. For more information, please contact the corresponding author.

## List of Abbreviations

AOFAS	American Orthopedic Foot & Ankle Society
BA	Böhler Angle
BMI	Body Mass Index
CI	Confidence Interval
CF	Calcaneal Fracture
CRIF	Closed Reduction and Internal Fixation
CRPS	Complex Regional Pain Syndrome
CT	Computed Tomography
IM	Intramedullary Nailing
IQR	Interquartile Range
MFS	Maryland Foot Score
NRS	Numerical Rating Scale
ORIF	Open Reduction and Internal Fixation
RR	Risk Ratio
SSI	Surgical Site Infection
